# Biochemical Characterization of CTX-M-15 from *Enterobacter cloacae* and Designing a Novel Non-β-Lactam-β-Lactamase Inhibitor

**DOI:** 10.1371/journal.pone.0056926

**Published:** 2013-02-21

**Authors:** Mohammad Faheem, Md Tabish Rehman, Mohd Danishuddin, Asad U. Khan

**Affiliations:** Medical Microbiology and Molecular Biology Laboratory, Interdisciplinary Biotechnology Unit, Aligarh Muslim University, Aligarh, UP, India; University of Cambridge, United Kingdom

## Abstract

The worldwide dissemination of CTX-M type β-lactamases is a threat to human health. Previously, we have reported the spread of *bla*_CTX-M-15_ gene in different clinical strains of *Enterobacteriaceae* from the hospital settings of Aligarh in north India. In view of the varying resistance pattern against cephalosporins and other β-lactam antibiotics, we intended to understand the correlation between MICs and catalytic activity of CTX-M-15. In this study, steady-state kinetic parameters and MICs were determined on *E. coli* DH5α transformed with *bla*_CTX-M-15_ gene that was cloned from *Enterobacter cloacae* (EC-15) strain of clinical background. The effect of conventional β-lactamase inhibitors (clavulanic acid, sulbactam and tazobactam) on CTX-M-15 was also studied. We have found that tazobactam is the best among these inhibitors against CTX-M-15. The inhibition characteristic of tazobactam is defined by its very low IC_50_ value (6 nM), high affinity (*K*_i_ = 0.017 µM) and better acylation efficiency (*k*_+2_/*K*′ = 0.44 µM^−1^s^−1^). It forms an acyl-enzyme covalent complex, which is quite stable (*k*_+3_ = 0.0057 s^−1^). Since increasing resistance has been reported against conventional β-lactam antibiotic-inhibitor combinations, we aspire to design a non-β-lactam core containing β-lactamase inhibitor. For this, we screened ZINC database and performed molecular docking to identify a potential non-β-lactam based inhibitor (ZINC03787097). The MICs of cephalosporin antibiotics in combination with this inhibitor gave promising results. Steady-state kinetics and molecular docking studies showed that ZINC03787097 is a reversible inhibitor which binds non-covalently to the active site of the enzyme through hydrogen bonds and hydrophobic interactions. Though, it’s IC_50_ (180 nM) is much higher than tazobactam, it has good affinity for CTX-M-15 (*K*_i_ = 0.388 µM). This study concludes that ZINC03787097 compound can be used as seed molecule to design more efficient non-β-lactam containing β-lactamase inhibitor that could evade pre-existing bacterial resistance mechanisms.

## Introduction

Antibiotic resistance in Gram-negative bacteria is a major health concern. It is principally observed due to the emergence of β-lactamase producers, which leads to the resistance against β-lactam antibiotic [1]. The β-lactamase enzymes are classified according to the scheme of Ambler into four classes, designated classes A to D, on the basis of their amino acid sequences, with classes A and C being the most frequently occurring among bacteria. Extended Spectrum β-lactamases (ESBLs) belong to molecular Amber class A or functional class 2be β-lactamases capable of conferring bacterial resistance to the penicillins, narrow-spectrum, expanded-spectrum and broad spectrum cephalosporins, and aztreonam, but not to cephamycins or carbapenems. Moreover, their activity is inhibited by β-lactamase inhibitors such as clavulanic acid, sulbactam and tazobactam [2], [3].

Plasmid-encoded ESBLs of the CTX-M type are increasingly being reported worldwide in Gram-negative bacteria and now account for most of the ESBL types found in the *Enterobacteriaceae* [4], [5]. They form a rapidly growing family that comprises more than 100 variants (http://www.lahey.org/studies) and are divided into five groups according to amino acid sequence identity, with different groups being prevalent in different countries [6], [7]. CTX-M ESBLs (derived its name from being highly active on CefoTaXime and isolated in Munich) are characterized by displaying greater hydrolytic activity against cefotaxime than against ceftazidime [8]. However, some clinical isolates have a significant degree of resistance to ceftazidime as well [9]. The first clinical strain producing CTX-M enzymes was found in Japan in 1993 with the characterization of the Toho-1 enzyme from an *E. coli* strain [10]. CTX-M-3 and its variants, CTX-M-15 were discovered in Poland, India, United Kingdom, Bulgaria, Romania and Turkey [9], [11], [12]. In India, CTX-M-15 is the most widespread ESBL and has been reported from six unrelated members of the family *Enterobacteriaceae* (four *E. coli* strains, one *K. pneumonia* strain, and one *E. aerogenes* strain) [9]. The widespread dissemination of CTX-M-15 has a significant impact on the treatment of hospital- and community-acquired infections caused by *E. coli* and other enteric bacilli [13]–[15].

**Figure 1:**
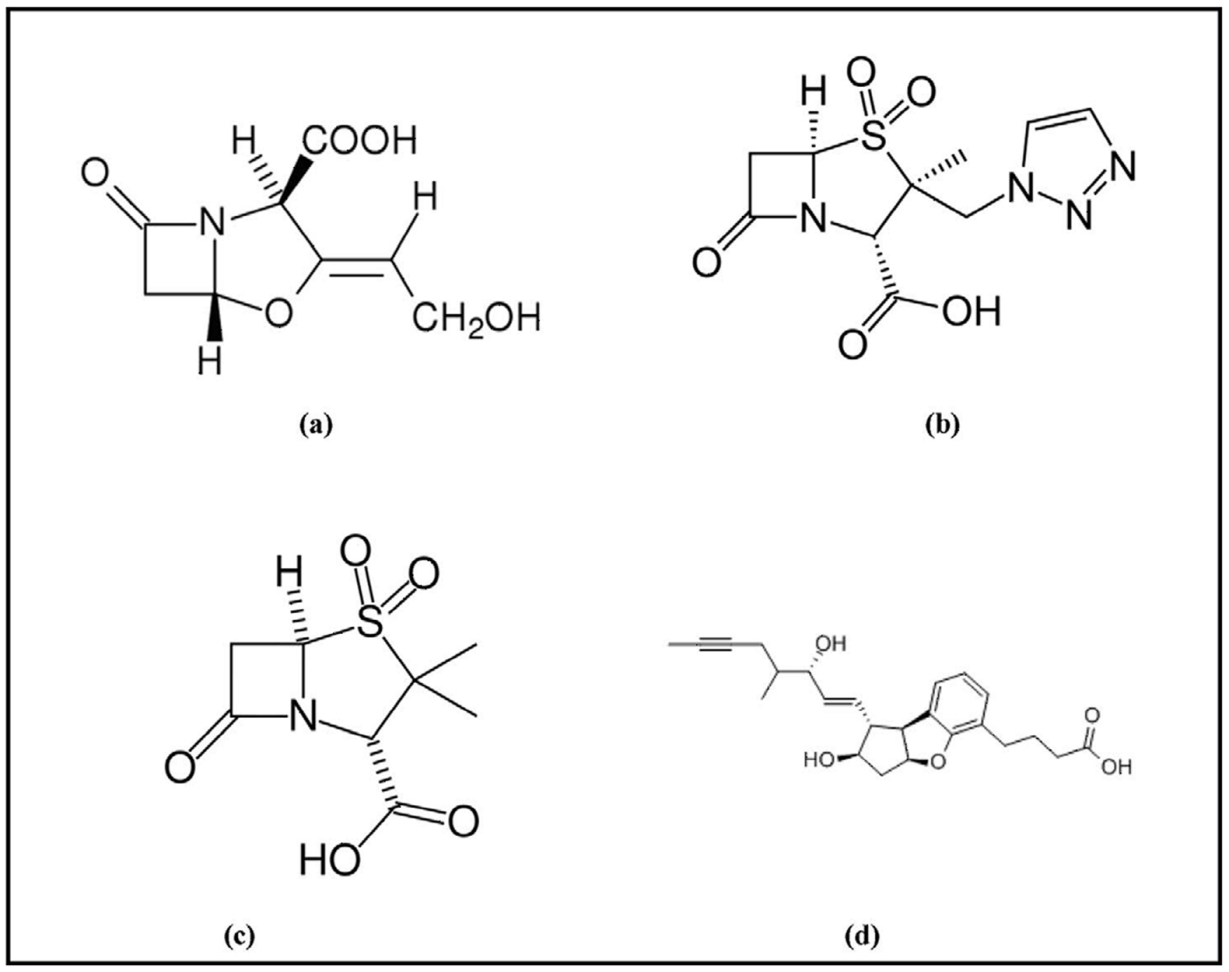
Chemical structure of β-lactamase inhibitors. Different inhibitors used in the study are (a) clavulanic acid, (b) tazobactam, (c) sulbactam, and (d) ZINC03787097.

Several β-lactamase inhibitors that are commonly used in combination with β-lactam antibiotics are clavulanic acid, tazobactam and sulbactam. Among class A enzymes, tazobactam is the most potent inhibitor followed by clavulanic acid and sulbactam [16]. The core structure of these inhibitors contains a β-lactam ring (Figure 1). Emergence of bacterial resistance against such inhibitors has been reported owing to the ability of bacteria to hydrolyse the β-lactam core of these inhibitors [17]–[19]. Porin channel mutation and overexpression of β-lactamases in the presence of β-lactam based inhibitor are other mechanisms that confer increasing resistance against such inhibitors [20]. Thus, there is an urgent need for the screening of novel inhibitors that do not contain a β-lactam core structure. Such inhibitors would not be hydrolyzed by wild type or mutant β-lactamases and would not be recognized by the ESBL producers [21]. Moreover, a novel non-β-lactam based inhibitor would not be affected by porin channel mutations, which prevent β-lactams from accessing their cellular targets. Furthermore, non-β-lactam based inhibitors would minimize the ability of bacteria to recruit existing resistance mechanisms, and bacteria would take a long time to develop novel mechanisms of resistance [22].

Previously, we have identified CTX-M-15 from *E. coli*, *E. cloacae*, *K. pneumoniae* and *A. Baumanii* from Aligarh hospital of north India and submitted their DNA sequences in Genbank [13], [23]. In the present study, blaCTX-M-15 from an Enterobacter cloacae clinical strain, EC-15 (Genbank accession no.: JN860195.1) was cloned and the enzyme was purified to homogeneity and an attempt has been made to understand the correlation between MICs and catalytic activity. This study also aimed to identify novel non-β-lactam core containing inhibitor and explore its mechanism of action.

## Materials and Methods

### Antibiotics and Other Chemicals

Ampicillin, Piperacillin, Cefazolin, Cefuroxime, Cefotaxime, Ceftriaxone, Ceftazidime, Cefepime and Aztreonam were purchased from Sigma chemical co. (St. Louis, MO), and Nitrocefin was purchased from Calbiochem (USA). Clavulanic acid, Sulabctam and Tazobactam were from Sigma-Aldrich (St. Louis, MO), while ZINC03787097 was purchased from Santa Cruz, India. IPTG (isopropyl-β-D-thiogalactopyranoside) was purchased from Roche (Basel, Switzerland). Other reagents and chemicals were of analytical grade. The structures of various inhibitors used in the present study were presented in figure 1.

### Bacterial Strains

*E. coli* DH5α and *E. coli* BL21 (DE3) were used for cloning and protein expression experiments, respectively. MICs were determined on *E. coli* DH5α transformed with cloned CTX-M-15 from *Enterobacter cloacae* clinical strain EC-15.

### Cloning and Sequencing

The plasmid DNA harbouring *bla*_CTX-M-15_ gene from clinical *E. cloacae* EC-15 strain (Genbank accession no.: JN860195.1), characterized in our lab, was extracted using Qiagen plasmid extraction kit, according to manufacturer’s instructions. The *bla*_CTX-M-15_ gene was amplified by PCR with the primers CTX-M-15-F (5′ ATATCATATGGTTAAAAAATCACTG 3′) containing Nde I site and CTX-M-15-R (5′ ATATAAGCTTTTACAAACCGTCGGTGAC 3′) containing Hind III site. The PCR conditions used were 95°C (30 s), 54°C (25 s), 72°C (40 s) and the reaction was carried out for 35 cycles. The PCR product does not contain the promoter region of the gene. The PCR product and pQE-2 (high copy cloning vector), were double digested with Nde I and Hind III, ligated and used to transform competent *E. coli* DH5α by heat shock method. Transformants harbouring *bla*_CTX-M-15_ gene were selected on LB agar plates containing ampicillin (100 µg/ml). The clones were confirmed by sequencing on both strands by standard procedures.

### CTX-M-15 β-lactamase Expression and Purification

To express and purify CTX-M-15 β-lactamase, the pQE-2 vector harbouring *bla*_CTX-M-15_ gene was transformed into competent *E. coli* BL21 (DE3) cells. A 5 ml overnight culture of these transformed cells in Luria-Bertani (LB) medium containing 100 µg/ml ampicillin was used to inoculate 1 litre of LB medium containing 100 µg/ml ampicillin. Bacteria were cultured at 37°C with shaking, until an optical density at 600 nm of 0.6 was reached. The culture was cooled and then transferred to 37°C, induced by 0.5 mM IPTG for three hours. The bacteria were collected by centrifugation and resuspended in 20 ml lysis buffer containing 50 mM Tris, pH 8.0, 300 mM NaCl and 0.1% β-mercaptoethanol per litre culture. The bacteria were ruptured by sonication, and the cell debris was removed by centrifugation at 12,000 rpm for 30 min. The cleared supernatant was loaded onto a Ni-NTA column, which was pre-equilibrated by lysis buffer, and washed with lysis buffer supplemented with 20 mM imidazole. Protein was eluted with PBS buffer containing 250 mM imidazole. Pure protein was obtained after dialysis in PBS (50 mM sodium phosphate, pH 8.0, containing 300 mM NaCl). Purity of the purified protein was estimated to be more than 95% as determined by a single band of 31 kDa on SDS-PAGE (Figure S1). The final protein concentrations were determined by using the molar extinction coefficient of 25, 440 M^−1^ cm^−1^ at 280 nm.

### Antibiotic Susceptibility Testing Based on MIC Analysis

The MICs for various antibiotics alone or in combination with various inhibitors (clavulanic acid, sulbactam, tazobactam and ZINC03787097) were determined by micro-dilution method [24] and the results were interpreted according to Clinical Laboratory Standards Institute (CLSI) guidelines [25]. Briefly, *E. coli* DH5α cells transformed with CTX-M-15 was treated with increasing concentrations of the antibiotics ranging from 0.5 to 2000 µg/ml in a series of two fold dilutions. The inhibitors clavulanic acid, sulbactam and tazobactam were used at a fixed concentration of 4 µg/ml, while ZINC03787097 was used at 20 µg/ml. The MIC was determined as the lowest concentration that totally inhibits visible bacterial growth.

### Determination of IC_50_

Various concentrations of clavulanic acid, tazobactam, sulbactam and ZINC03787097 were pre-incubated with the purified CTX-M-15 for 5 mins at 30°C before the rate of nitrocefin (100 µM) hydrolysis was measured [26], [27]. The 50% inhibitory concentration (IC_50_) was determined as the concentration of the inhibitor that inhibited hydrolytic activity of the enzyme by 50%.

### Steady-state Kinetics Experiments

The steady-state kinetics parameters of CTX-M-15 were determined on the following antibiotics: Ampicillin (Δε_235_ = −900 M^−1^ cm^−1^), Piperacillin (Δε_232_ = −1640 M^−1^ cm^−1^), Nitrocefin (Δε_486_ = +15,000 M^−1^ cm^−1^), Cefazolin (Δε_320_ = +1067 M^−1^ cm^−1^), Cefuroxime (Δε_262_ = −8,540 M^−1^ cm^−1^), Cefotaxime (Δε_264_ = −7,250 M^−1^ cm^−1^), Ceftazidime (Δε_265_ = −10,300 M^−1^ cm^−1^), Cefepime (Δε_267_ = −9,120 M^−1^ cm^−1^) and Aztreonam (Δε_318_ = −650 M^−1^ cm^−1^). The inhibitors used were Clavulanic acid, Sulabctam, Tazobactam and ZINC03787097.

Hydrolysis of β-lactam antibiotics was detected by monitoring the variation in the absorbance due to cleavage of β-lactam ring in 50 mM phosphate buffer, pH 7.0 [28]. All the measurements were taken in triplicate on Shimadzu UV-VIS spectrophotometer (UV-1800). The reaction was performed in a total volume of 500 µl at 30°C. For dilution of the enzyme and to prevent denaturation, BSA was added to a final concentration of 20 µg/ml.

The kinetic parameters (*k*_cat_ and *K*_m_) for the hydrolysis of good substrates (nitrocefin, ampicillin, piperacillin, cefazolin, cefuroxime, cefotaxime, and ceftriaxone) were determined by measuring the initial rate of antibiotic hydrolysis and by using Michaelis-Menten equations 1 and 2 [29].
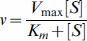
(1)

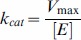
(2)where, v and V_max_ are the initial and maximum velocity of hydrolysis, respectively, [S] is the concentration of the substrate used, [E] is the enzyme concentration in the reaction, and *K*_m_ is the Michaelis-Menten constant.

For poor substrates (ceftazidime, cefepime and aztreonam) and inhibitors (clavulanic acid, sulbactam, tazobactam, and ZINC03787097), the *k*_cat_ values were determined from the initial rates calculated at saturating substrate concentrations (i.e. under zero^th^ order kinetics), and the *K*_m_ values were determined as competitive inhibition constant (*K*_i_) in a competition experiment between tested antibiotic/inhibitor and 100 µM nitrocefin used as reporter substrate, and the result was analysed according to equation 3 [28].
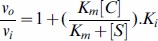
(3)where, v_o_ and v_i_ represent the initial rate of nitrocefin hydrolysis in the absence and presence of the poor substrate, respectively; [*C*] is the concentration of poor substrate/inhibitor; and *K*_m_ and [S] are the Michaelis-Menten constant and concentration of nitrocefin, respectively.

#### Evaluation of enzyme inhibition kinetic results

The interaction of β-lactamases with inhibitors is represented by a simple three-step acylation-deacylation mechanism [30]–[32] as shown:
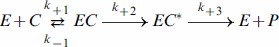
(4)where, E is enzyme, C is inhibitor, EC is non-covalent Henri-Michaelis complex, EC* is acyl-enzyme intermediate and P is inactive degradation product of the substrate. The parameters *k*_+2_, *k*_+3_ and *K′* are the first-order acylation and deacylation constants, and the dissociation constant of the Henri-Michaelis complex, respectively. The steady-state kinetic parameters for the above equation are given by the following equations:



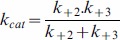
(5)

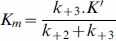
(6)

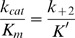
(7)

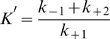
(8)


The values of the first-order rate constant (*k*_i_) characterizing the rate of EC* accumulation was calculated by measuring the hydrolysis of nitrocefin (reporter substrate) at different inhibitor concentrations according to the following equation [28]:
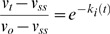
(9)


where, *v*_o_, *v*_t_ and *v*_ss_ are the rates of utilization of the reporter substrate at times 0 and t, and after the steady-state has been established, respectively.

The individual parameters, *k*_+2,_ *k*_+3_ and *K′*, were derived from the dependence of *k*_i_ upon inhibitor concentration, [C], on the basis of the following equation:
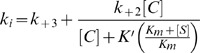
(10)


where, *K*_m_ and [S] are the Michaelis-Menten constant and concentration of the reporter substrate, nitrocefin.

When the concentration of inhibitor [C] is well below *K′*, *k*_i_ varies linearly the above equations is simplified as:
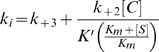
(11)


The *k*_+2_/*K′* value is then obtained from the slope of the line, whereas *k*_+3_ is given by the intercept at [C] = 0. Conversely, if [C] is much larger than *K′* (*K*_m_+[S])/*K*_m_, then *k*_i_ = *k*_+2_+*k*_+3_, and is independent of [C] [28].

### Mechanism of Action of ZINC03787097

The mode of action of ZINC03787097 was determined by monitoring the hydrolysis of nitrocefin (10–100 µM) by CTX-M-15 (0.50 nM) in the presence of different concentration of ZINC03787097 (0.25–0.75 µM) in 50 mM phosphate buffer, pH 7.0 at 30°C [28]. All the measurements were taken in triplicate on Shimadzu UV-VIS spectrophotometer (UV-1800). The result was analysed from Lineweaver-Burg plot.

### Stability of ZINC03787097 in the Presence of CTX-M-15

The UV absorption spectrum (320-210 nm) of ZINC03787097 (100 µM) alone or after 3600 seconds incubation with CTX-M-15 (10 nM) was measured in 50 mM phosphate buffer, pH 7.0 at 30°C on Shimadzu UV-VIS spectrophotometer (UV-1800). The stability of ZINC03787097 in the presence of CTX-M-15 was monitored by measuring the absorbance for 3600 seconds at 283 nm and 222 nm.

### *In silico* Modelling and Molecular Docking

Protein sequence of CTX-M-15 was retrieved from NCBI (ID: ABM88811). Three dimensional structure of this protein was built by using the template (pdb:1IYS), having 86% identity. The selected model was refined by performing energy minimization through a CHARMm force field [33] with Dependent Dielectric implicit solvent model and conjugates gradient method. This process was carried out until the average absolute derivative of co-ordinates with respect to energy fell below 0.1 kcal Å^−1^. Molecular docking of known β-lactamase inhibitors (clavulanic acid, sulbactam and tazobactam) along with screened inhibitor (ZINC03787097) from ZINC database [34] was performed by using GOLD (Genetic Optimization for Ligand Docking) 5.0 program [35]. GOLD fitness score was used as a parameter to define the efficiency of selected inhibitors.

## Results

### Antibiotic Susceptibility Testing Based on MIC Analysis

The MICs of the β-lactam antibiotics alone or in combination with different inhibitors (clavulanic acid, sulbactam, tazobactam and ZINC03787097) were determined on *E. coli* DH5α harbouring *bla*_CTX-M-15_ gene from *Enterobacter cloacae* isolate (EC-15) of clinical background and the results are presented in table 1. Very high MICs (500–1000 µg/ml) were obtained for ampicillin, piperacillin, cefazolin, cefuroxime, cefotaxime and ceftriaxone, indicating that the studied strain is highly resistant to these antibiotics. The MICs of ceftazidime, cefoxitin, aztreonam and cefepime (4, 16, 16 and 125 µg/ml, respectively) were moderate, but still is in the resistant range.

**Table 1:** MICs of β-lactams alone or in combination with inhibitors for *E. coli* DH5α transformed with recombinant *bla*_CTX-M-15_ from *Enterobacter cloacae*.

Antibiotic+Inhibitors	MIC(µg/ml)	
	DH5α(pQE-2-CTX-M-15)^a^	DH5α(Null Plasmid)
Piperacillin	>1024	1
Ampicillin	>1024	2
Ampicillin+Clavulanic Acid	512	2
Ampicillin+Sulbactam	>1024	2
Ampicillin+Tazobactum	>1024	4
Ampicillin+ZINC03787097	>1024	4
Cefazolin (I)	>1024	2
Cefuroxime (II)	>1024	1
Cefotaxime (III)	512	0.25
Cefotaxime (III)+ClavulanicAcid	32	0.25
Cefotaxime (III)+Sulbactam	256	0.25
Cefotaxime (III)+Tazobactum	64	0.25
Cefotaxime(III)+ZINC03787097	256	0.25
Ceftriaxone (III)	>1024	0.06
Ceftazidime (III)	4	0.25
Ceftazidime (III)+Clavulanic Acid	2	0.12
Ceftazidime (III)+Sulbactam	2	0.25
Ceftazidime (III)+Tazobactum	2	0.25
Ceftazidime(III)+ZINC03787097	2	0.25
Cefepime (IV)	128	0.25
Cefepime (IV)+Clavulanic Acid	16	0.02
Cefepime (IV)+Sulbactam	64	0.02
Cefepime (IV)+Tazobactum	8	0.02
Cefepime (IV)+ZINC03787097	64	0.12
Cefoxitin	16	2
Cefoxitin+Clavulanic Acid	2	1
Cefoxitin+Sulbactam	4	1
Cefoxitin+Tazobactum	2	1
Cefoxitin+ZINC03787097	2	1
Aztreonam	16	0.25

^a^ cloned from *Enterobacter cloacae* strain EC-15 (Genbank accession no.: JN860195.1).

Generation of cephalosporin antibiotics are given in parenthesis.

Clavulanic acid, sulbactam and tazobactam were used at 4µg/ml, while and ZINC03787097 was used at 20 µg/ml.

The efficacy of antibiotic-inhibitor combination was also studied on DH5α transformed with *bla*_CTX-M-15_ gene (Table 1). It was found that none of the inhibitors considered in the study were able to reduce the MIC of ampicillin. The MIC of third generation cephalosporin cefotaxime was lowered 16, 2, 8 and 2 fold when the antibiotic was used in combination with clavulanic acid, sulbactam, tazobactam and ZINC03787097, respectively. The MIC of ceftazidime in combination with various inhibitors was reduced 2-folds to 2 µg/ml, and bought down to the susceptible range. Similarly, the MIC of cefepime was reduced to 2–16 folds in the presence of various inhibitors. The greatest effect of inhibitors on MIC was observed when they are given in combination with cefoxitin. The MIC was reduced from 16 µg/ml to 4 µg/ml for cefoxitin-sulbactam combination, and to 2 µg/ml in the case of cefoxitin with clavulanic acid, tazobactam and ZINC03787097.

### IC_50_ Determination

**Figure 2:**
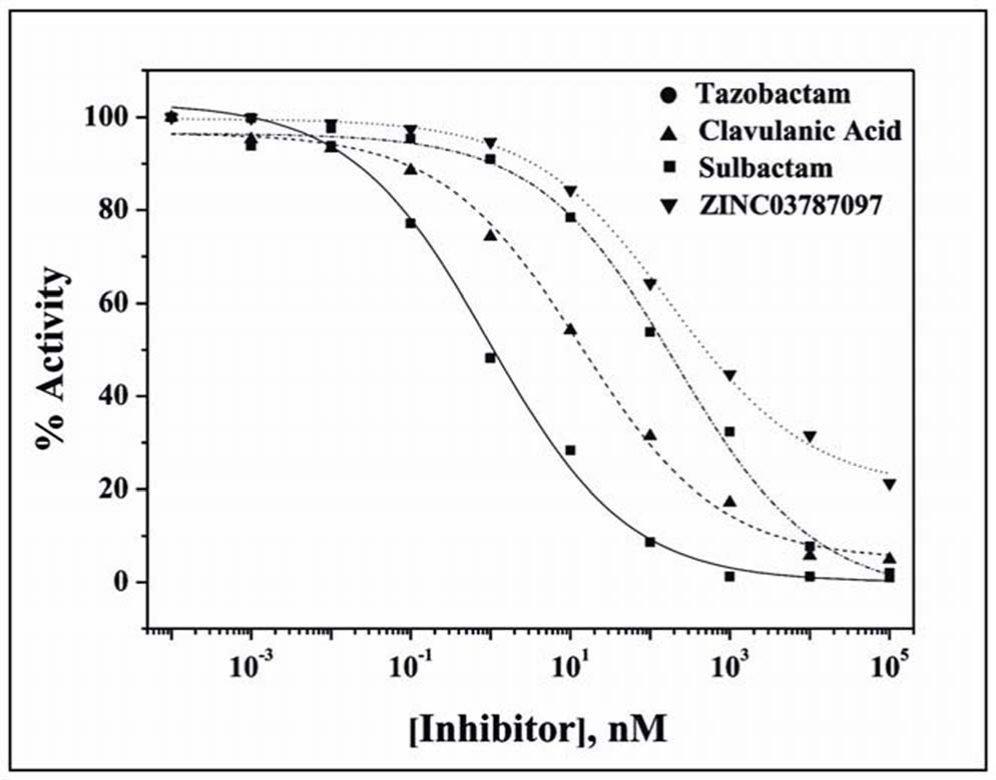
Determination of IC_50_ values for different inhibitors. The residual activity of CTX-M-15 after 5 minutes pre-incubation with varying concentrations of different inhibitors as monitored by the hydrolysis of 100 µM nitrocefin.

The IC_50_ values are appropriate for accessing the potency of an inhibitor or comparing the potential of different inhibitors under properly controlled experiments. The IC50 values were determined by exposing CTX-M-15 to different inhibitors, namely clavulanic acid, sulbactam, tazobactam and ZINC03787097 for 5 mins and measuring the percent residual enzyme activity on nitrocefin (100 µM). The result is presented in figure 2 and table 2.

**Table 2:** Concentration of the inhibitors required to reduce the enzyme activity by 50%.

Enzyme	IC_50_ (nM)			
	Clavulanic acid	Tazobactam	Sulbactam	ZINC03787097
CTX-M-15	14	1	212	180

Among the four inhibitors studied, tazobactam displayed lowest IC50 value (1 nM), closely followed by clavulanic acid (14 nM). Comparatively, the IC_50_ value of ZINC03787097 is quite high (180 nM), but it is similar to that of sulbactam (212 nM).

### Steady-state Kinetic Parameters Determination

The steady-state kinetics of the purified CTX-M-15 was carried out on nitrocefin, ampicillin, piperacillin, cefazolin, cefuroxime, cefotaxime, ceftriaxone, ceftazidime, cefepime and aztreonam. The representative Michaelis-Menten plots are shown in figure S2, and the deduced kinetic parameters (*k*_cat_, *K*_m_ and *k*_cat_ */K*_m_) are summarized in table 3.

**Table 3:** Steady-state kinetic parameters of CTX-M-15 from *Enterobacter cloacae*.

Antibiotic	CTX-M-15^a^		
	*k*_cat_(s^−1^)	*K*_m_ or *K*_i_(µM)	*k*_cat_/*K*_m_(µM^−1^ s^−1^)
Nitrocefin	582±20	35±4	16.6
Ampicillin	57±3	15±2	3.8
Piperacillin	58±4	32±5	1.8
Cefazolin (I)	169±6	59±6	2.9
Cefuroxime (II)	87±2	29±2	3.0
Cefotaxime (III)	222±7	60±3	3.7
Ceftriaxone (III)	82±5	33±2	2.5
Ceftazidime (III)	1.51±0.03	1209±91	0.0012
Cefepime (IV)	17±3	548±25	0.03
Aztreonam	2.38±0.06	21±3	0.11

^a^ cloned from *Enterobacter cloacae* strain EC-15 (Genbank accession no.: JN860195.1).

Generations of cephalosporin antibiotics are given in parenthesis.

Kinetic parameters of the purified CTX-M-15 β-lactamase revealed a hydrolytic profile that is a characteristic of molecular class A CTX-M type β-lactamases and had activity against restricted as well as expanded spectrum β-lactam antibiotics [36]. The enzyme showed good affinity (*K*_m_ in 15–60 µM range) for all the studied antibiotics except ceftazidime and cefepime, for which *K*_m_ was 1209 and 548 µM, respectively. Low affinity along with poor catalytic activity (1.51 and 17 s^−1^ for ceftazidime and cefepime, respectively) made CTX-M-15 inefficient in hydrolyzing these antibiotics. The representative catalytic efficiency for ceftazidime and cefepime are 0.0012 and 0.03 µM^−1 ^s^−1^, respectively. It was also clear from table 3 that the enzyme had high catalytic efficiency (*k*_cat_/*K*_m_ in 2–4 µM^−1 ^s^−1^ range) for ampicillin, piperacillin, cefazolin, cefuroxime, cefotaxime and ceftriaxone. The highest catalytic activity and efficiency was observed for nitrocefin, the values being 582 s^−1^ and 16.6 µM^−1 ^s^−1^, respectively. The study clearly indicates that CTX-M-15 had some residual activity, although relatively low, on cefepime and ceftazidime, which was further confirmed from MICs of *bla*_CTX-M-15_ expressing *E. coli* DH5α transformed cells.

**Figure 3:**
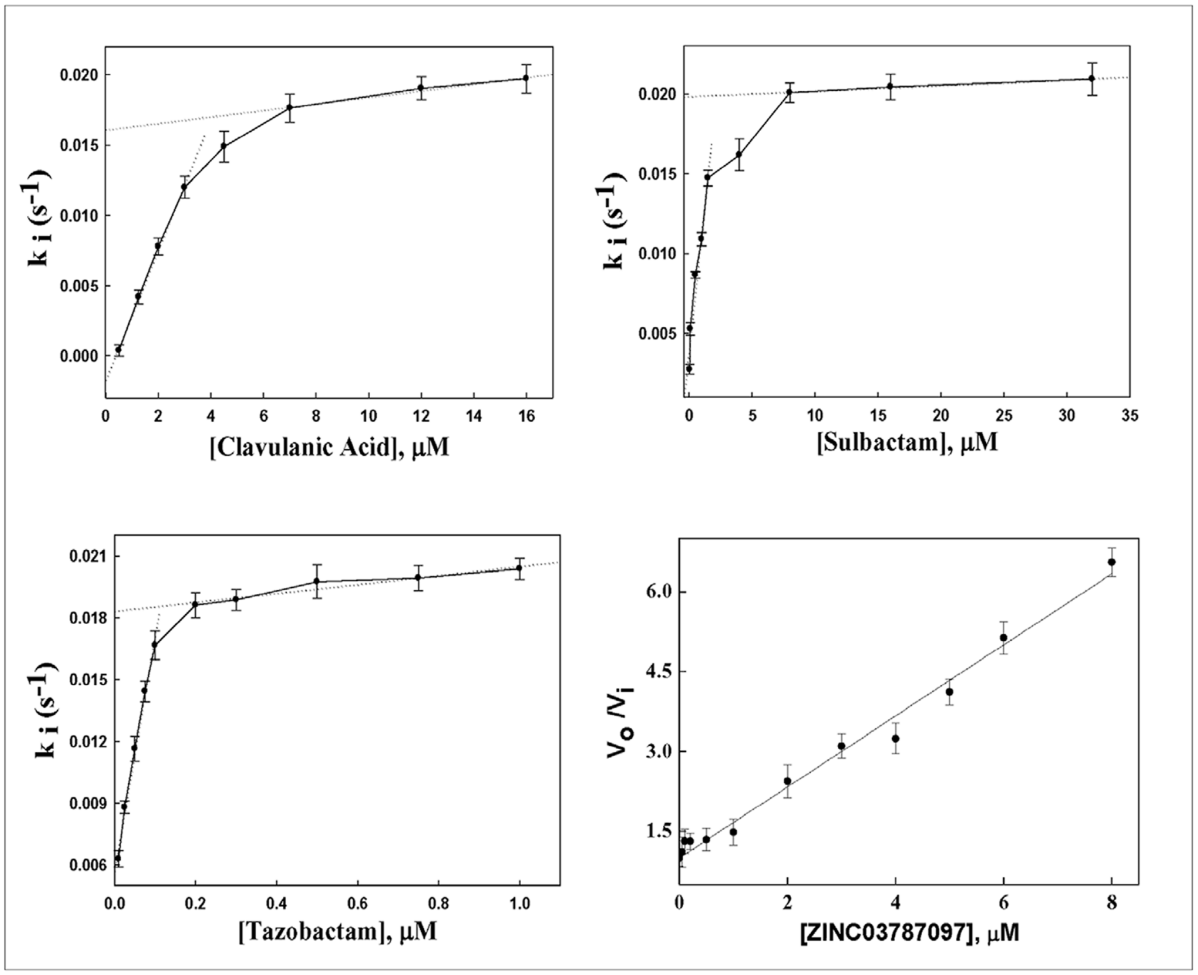
Effect of various inhibitors on the kinetics of CTX-M-15. Variation of inactivation rate constant (*k*_i_) versus different inhibitor concentrations for CTX-M-15 from clinical *Enterobacter cloacae* strain EC-15. The dotted line represents the best fit (linear regression) among the data points.

The enzyme kinetics parameters of CTX-M-15 in the presence of various inhibitors are presented in table 4. CTX-M-15 was efficiently acylated by clavulanic acid, sulbactam and tazobactam, and individual kinetic parameters were computed. The catalytic activity of CTX-M-15 on clavulanic acid, sulbactam and tazobactam (measured as *k*_+3_) was determined as 0.0019, 0.0038 and 0.0057 s^−1^, respectively. Moreover, the affinity (*K*_i_) of CTX-M-15, determined at steady-state, was found to be highest for tazobactam (0.017 µM) as compared to clavulanic acid (0.099 µM) and sulbactam (0.062 µM). The calculated *K*_i_ values (*K*_i_ = *k*_+3_ *K*/*k*_+2_) for clavulanic acid, sulbactam and tazobactam were 0.106, 0.131 and 0.013 µM, respectively, and was in good agreement with the experimentally determined values (Table 4). The *K*_i_ of CTX-M-15 for ZINC03787097 was found to 0.388 µM, which was much higher than the other inhibitors. The acylation efficiency (*k*_+2_/*K*) of the enzyme was determined from the slope of a plot of ki values versus different inhibitor concentrations (Figure 3). It was found to be highest for tazobactam (0.44 µM^−1^ s^−1^), followed by sulbactam (0.029 µM^−1^ s^−1^) and clavulanic acid (0.018 µM^−1^ s^−1^). The high acylation efficiency and low deacylation efficiency (measured as *k*_+3_) made tazobactam best among mechanism based inhibitors.

**Table 4:** Effect of various inhibitors on the steady-state kinetic parameters of CTX-M-15.

Inhibitors	*K*_i_(µM)	*k*_cat_/*K*_m_ or *k*_+2_/*K′* (µM^−1^ s^−1^)	*k*_cat_ or *k*_+3_(s^−1^)	*k*_+2_ x 10^−2^(s^−1^)	*K′*(µM)
Clavulanic acid	0.099 (0.106*)	0.018	0.0019	1.3	0.72
Sulbactam	0.062 (0.131*)	0.029	0.0038	1.6	0.55
Tazobactam	0.017 (0.013*)	0.44	0.0057	1.3	0.03
ZINC03787097	0.388	ND	ND	–	–

SD values were below 10%.

ND = not determinable.

calculated as *K*_i_ = *k*_+3_ *K*/*k*_+2_.

### Mechanism of Action of ZINC03787097

Figure S3 shows Lineweaver-Burg plot for the hydrolysis of different concentrations of nitrocefin by CTX-M-15 in the presence of various ZINC03787097 concentrations. The values of *K*_m_ for nitrocefin hydrolysis in the presence of 0.00, 0.25, 0.50 and 0.75 µM ZINC03787097 were 42, 67, 94 and 96 µM, respectively. On the other hand, the value of V_max_ ( = 3.22×10^−7^ M s^−1^) was similar in all cases. Thus, it is clear that ZINC03787097 inhibited CTX-M-15 reversibly through competitive inhibition.

### Stability of ZINC03787097 in the Presence of CTX-M-15

The absorption spectrum of ZINC03787097 gave characteristic peaks at 283 nm and 222 nm even after pre-incubation with CTX-M-15 for 3600 seconds. Moreover, no detectable hydrolysis of ZINC03787097 by CTX-M-15, as monitored by measuring the change in absorbance at 283 nm and 222 nm, was detected even after 3600 seconds incubation (Figure S4).

### *In-silico* Screening of non-β-lactam Based β-lactamase Inhibitor and its Evaluation

Fitness score from GOLD program of clavulanic acid, sulbactam and tazobactam was 34.89, 34.36 and 41.66 respectively whereas fitness score of screened compound (ZINC03787097) was 47.56. It is clear that the compound ZINC03787097 was found to have highest fitness score as compared to that of known inhibitors of CTX-M-15.

**Figure 4:**
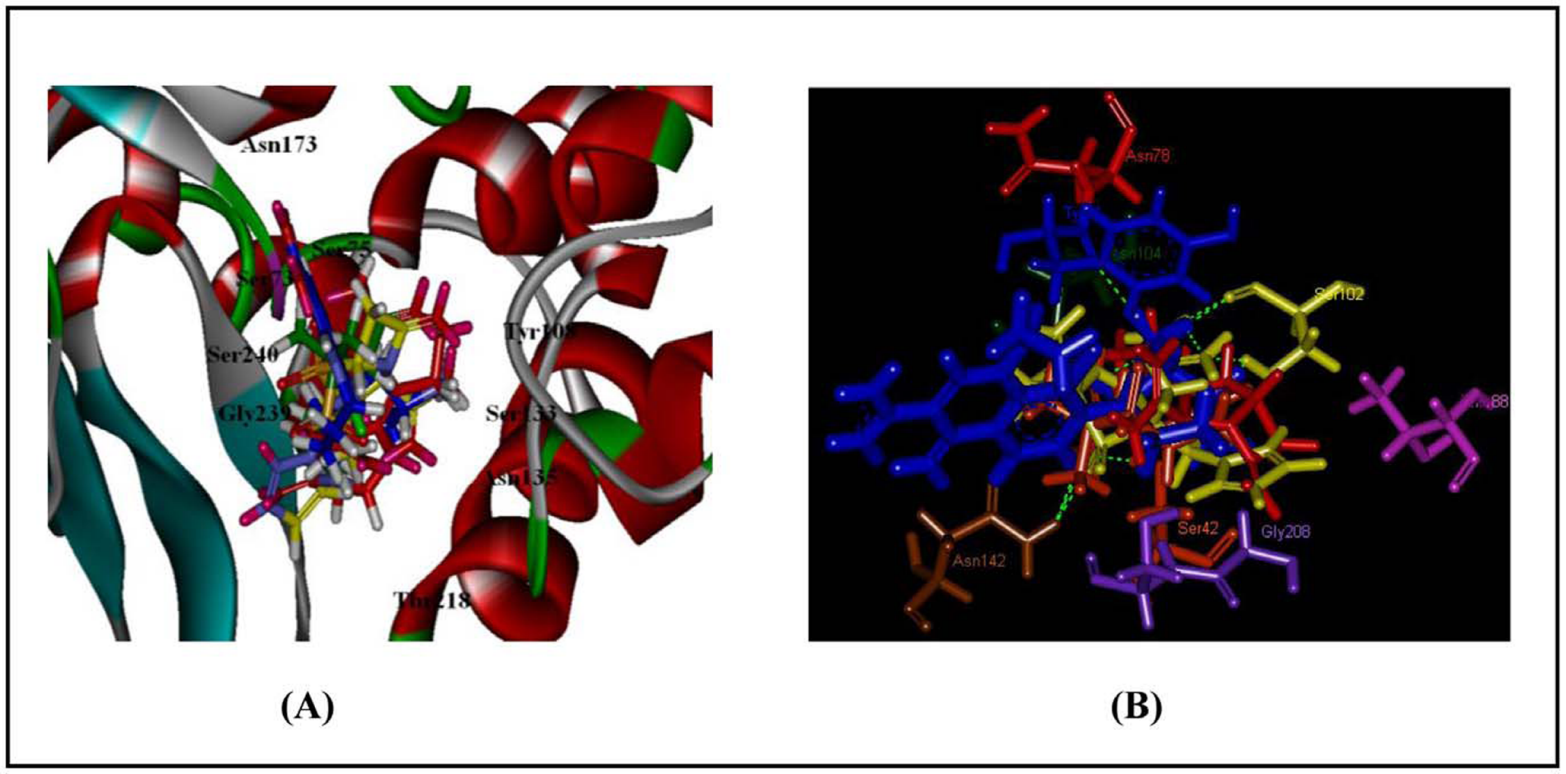
Molecular docking of various inhibitors at the active site of CTX-M-15. Panel (A) shows molecular docking of clavulanic acid (red), sulbactam (green), tazobactam (yellow) and screened compound ZINC03787097 (blue) at the active site of CTX-M-15 from clinical strain of *Enterobacter cloacae* (EC-15). Panel (B) represents close view of interacting amino acids in which dashed lines represent hydrogen bonds. Ambler positions of amino acids are Ser-73 (Ser-42), Tyr-108 (Tyr-77), Ser-133 (Ser-102), Asn-135 (Asn-104), Asn-173 (Asn-141), Gly-239 (Gly-208), Ser-240 (Ser-209), Thr-218 (Thr-188).

Three hydrogen bonds were observed in between the novel compound and CTX-M-15. Amino acids Tyr-108, Ser-133 and Asn-173 were involved in hydrogen bonding interactions (Figure 4). Comparatively, lesser hydrogen bond interactions were observed in the case of CTX-M complexed with known inhibitors. Moreover, amino acids which are important for catalytic activity (Ser-73, Ser-75, Tyr-108, Ser-133, Asn-135, Thr-218, Gly-239 and Ser-240) were found to interact with ZINC03787097 through hydrophobic interactions (Figure 4).

## Discussion

The treatment of bacterial infections with antibiotics is one of the key concepts of human medicine. However, the effectiveness of antibiotics has become limited owing to an increase in bacterial antibiotic resistance, which represents a global health problem with a strong social and economic impact [37], [38]. The resistance to β-lactam antibiotics, including penicillins and cephalosporins, which are amongst the most widely used class of antibiotics, is a serious problem and needs an immediate attention. Several factors contribute to this resistance mechanism like (i) mutations in the target of these drugs i.e. penicillin binding proteins involved in cell-wall biosynthesis [39], (ii) deletion and/or modification of the porin channels through which the drugs diffuse [40], (iii) expression of pumps that export the drugs out of the bacterial cells [41], and (iv) overexpression of β-lactamases in the presence of antibiotics [42], [43]. The most widespread resistance mechanism remains the expression of β-lactamase enzymes, which inactivate the β-lactam antibiotics by hydrolyzing their β-lactam ring [44], [45].

In India, CTX-M-15 is the most widely disseminated ESBL in hospital and community settings. It is highly active on cefotaxime and increasing resistance has been observed against ceftazidime as well, suggesting that the enzyme is evolving as a result of ceftazidime selection pressure [1], [9]. In this study, we have cloned *bla*_CTX-M-15_ from an *Enterobacter cloacae* strain (EC-15) of clinical origin (Genbank accession no.: JN860195.1) and the enzyme was purified to homogeneity (Figure S1). The resistance pattern was observed by determining MICs of common β-lactam antibiotics on *E. coli* DH5α transformed with cloned bla_CTX-M-15_ gene and steady-state enzyme kinetics of CTX-M-15 with β-lactam antibiotics and inhibitors. This is the first study that reported detailed CTX-M-15 inhibition kinetics mediated by conventional β-lactamase inhibitors such as clavulanic acid, sulbactam and tazobactam. An attempt has also been made to identify novel non-β-lactam core containing β-lactamase inhibitors by performing virtual screening of ZINC database. One such inhibitor, ZINC03787097, was identified and characterized by MICs, IC_50_ and enzyme kinetics.

MICs analysis showed that *Enterobacter cloacae* clinical isolate EC-15 is highly resistant to narrow as well as extended spectrum β-lactam antibiotics such as ampicillin, penicillin and various cephalosporins including cefotaxime and ceftazidime (Table 1). The MIC values obtained in our study against different β-lactam antibiotics were in excellent agreement with an earlier report [46]. Although, the MIC values for ceftazidime, cefepime and aztreonam were comparatively lower, *E. coli* DH5α cells expressing cloned *bla*_CTX-M-15_ were also resistant to these antibiotics. The lower MICs for ceftazidime, cefepime and aztreonam observed in our study is due to less selection pressure on *bla*_CTX-M-15_ harbouring bacterial population, as the consumption of these antibiotics in the treatment of bacterial infections is low. The MIC results were well supported by steady-state kinetics data, which revealed that CTX-M-15 possessed high activity against various cephalosporins including cefotaxime, and had ceftazidime, cefepime and aztreonam cleavage activity as well (Figure S2, Table 2). The broad spectrum β-lactamase activity of CTX-M-15 on various antibiotics can be explained by the replacement of Asp-240 by Gly-240, as it was evolved from CTX-M-3. Glycine at position 240 improved the substrate spectrum to include ceftazidime and aztreonam as well. The mutation of the active-site (but non-catalytic) Asp-240 to Gly-240 abolished the interaction between Asp-240 and Asn-270, which in turn improved the flexibility of the C-terminal *β*3 strand of the enzyme, and thus expanded the substrate spectrum to include ceftazidime and aztreonam [47]. The improvement in ceftazidime hydrolysis due to Asp240Gly substitution in CTX-M enzymes had also been reported in CTX-M-27 and CTX-M-16, which were evolved from CTX-M-9 and CTX-M-14, respectively [48], [49]. Overall, the kinetic data together with MIC values clearly showed that the β-lactamase included in this study is a typical CTX-M-15 enzyme, effective in hydrolyzing β-lactam antibiotics. It possessed high cefotaxime hydrolyzing capability and is also able to hydrolyze ceftazidime as well.

The combination of antibiotics with β-lactam based inhibitors (such as clavulanic acid, sulbactam and tazobactam) is very effective in controlling the widespread dissemination of β-lactamases. In this study, the effect of β-lactamase inhibitors on CTX-M-15 was studied by measuring MICs of various β-lactam antibiotics in combination with different inhibitors and by determining enzyme inhibition kinetic parameters. It was found that tazobactam reduced the MICs of β-lactam antibiotics to 2–16 folds and was the most potent mechanism based inhibitor of CTX-M-15 followed by clavulanic acid and sulbactam. Moreover, we found that the MICs of antibiotics of cephalosporin group were also reduced in the presence of ZINC03787097 to a level observed in the case of antibiotic-sulbactam combination (Table 1). The major cause of concern to the currently available β-lactam-β-lactamase inhibitor formulations is the emergence of resistance to amoxicillin-clavulanate and ticarcillin-clavulanate in isolates of *E. coli* and *K. pneumoniae* [50]–[53]. The emergence of this phenotype might be resulted from the production of β-lactamases that are not susceptible to inhibitors (e.g., Amp C from *Enterobacter* spp, or *P. aeruginosa* or metallo-β-lactamases) or the over-expression of β-lactamases due to mutations in the promoter region of the gene and/or high copy number of plasmids carrying the *bla* gene, as in the case of TEM-1 [54]–[56]. Thus, ZINC03787097 in combination with antibiotics of cephalosporin group could be used as an alternative to the conventional antibiotic-inhibitor combinations.

The enzyme inhibition kinetics (Figure 3, Table 4) revealed that CTX-M-15, like other class A serine β-lactamases, hydrolyses β-lactam antibiotics by a two step mechanism: acylation and deacylation. The acylation step is mediated by the nucleophilic attack of an active-site serine O-atom on the carbonyl carbon of the β-lactam ring, resulting in formation of covalent acyl-enzyme. On the other hand, deacylation is achieved by the activation of a water molecule by Glu-166, which acts as a general base. The resulting hydroxide then attacks the acyl carbonyl and hydrolyzes the acyl-enzyme intermediate [57]. In the case of interaction with β-lactam antibiotics, the deacylation rate is very high and the active enzyme is rapidly regenerated, whereas, interaction with β-lactam inhibitors results in an acyl-enzyme complex of substantial stability. We found that tazobactam had greatest affinity among conventional inhibitors for CTX-M-15 and displayed highest acylation efficiency and lowest deacylation efficiency. Moreover, the low *K*_i_ value (0.388 µM) and negligible hydrolysis (*k*_cat_) of inhibitor ZINC03787097 was observed (Figure S4). It competes with β-lactam antibiotic substrates for the active site of CTX-M-15 (competitive inhibition) and binds tightly through hydrogen bonding and hydrophobic interactions with the key residues involved in catalysis (Figure 4).

### Conclusion

Our study concludes that ZINC03787097 is a novel non-β-lactam inhibitor that complements the active site of the enzyme and interacts with key residues involved in β-lactam recognition and hydrolysis. Unlike conventional β-lactamase inhibitors, it binds to the active site through non-covalent interactions (hydrogen bonding and hydrophobic interactions). The advantage of using such inhibitors is that β-lactamase producers cannot recruit the existing resistance mechanisms against them. Our kinetic studies together with IC_50_ values and promising MICs results (when used in combination with antibiotics of cephalosporin group) indicate that ZINC03787097 is a suitable lead molecule for the development of more potent non-β-lactam based β-lactamase inhibitors.

## Supporting Information

Figure S1 **Cloning and purification of CTX-M-15.** Panel (A) shows the vector used for cloning and expression of *bla*_CTX-M-15_ gene. Panel (B) is the SDS-PAGE of the purified CTX-M-15. Lane 1 and 2 are overexpressed total cell protein and purified protein, respectively. The single band represents molecular mass of 31 kDa.
(TIF)

Figure S2 **Steady-state kinetics of CTX-M-15 on various β-lactam antibiotics.** The hydrolysis of good substrates was analysis by plotting graphs according to Michaelis-Menten equation and the kinetic parameters (*k*_cat_ and *K*_m_) were determined. For poor substrates, *K*_m_ was determined as *K*_i_ in a competition experiment using 100 µM nitrocefin as reporter substrate, and *k*_cat_ was estimated from the initial rates observed under saturating substrate concentrations (zero^th^ order reaction kinetics). Each point on these graphs is the mean value obtained from three independent experiments.
(TIF)

Figure S3 **Lineweaver-Burg plot.** The mechanism by which ZINC03780797 inhibits CTX-M-15 as determined by monitoring the hydrolysis of different concentrations of nitrocefin (10–100 µM) by 0.50 nM CTX-M-15 in the presence of varying ZINC03787097 concentrations: (a) 0 µM, (b) 0.25 µM, (c) 0.50 µM, and (d) 0.75 µM.
(TIF)

Figure S4 **Stability of ZINC03787097 in the presence of CTX-M-15.** Panel (A) shows the absorbance spectra of ZINC03787097 before and after 3600 seconds pre-incubation with CTX-M-15 at 30°C. Panel (B) shows the hydrolysis of ZINC03787097 by CTX-M-15 at 30°C monitored by measuring the change in absorbance at 283 nm and 222 nm.
(TIF)
