# Correction: Biochemical Characterization of CTX-M-15 from *Enterobacter cloacae* and Designing a Novel Non-β-Lactam-β-Lactamase Inhibitor

**DOI:** 10.1371/annotation/049bf1aa-d866-471f-95c1-5939d4461f8c

**Published:** 2013-12-30

**Authors:** Mohammad Faheem, Md Tabish Rehman, Mohd Danishuddin, Asad U. Khan

Article republished due to omission of equations in previous edition. 

