# Supplementary figures and images for: Biochemical Characterization of CTX-M-15 from Enterobacter cloacae and Designing a Novel Non-β-Lactam-β-Lactamase Inhibitor
Source: PLoS One. 2013 Feb 21;8(2):e56926. doi: 10.1371/journal.pone.0056926 (PMC3578935; doi:10.1371/journal.pone.0056926)

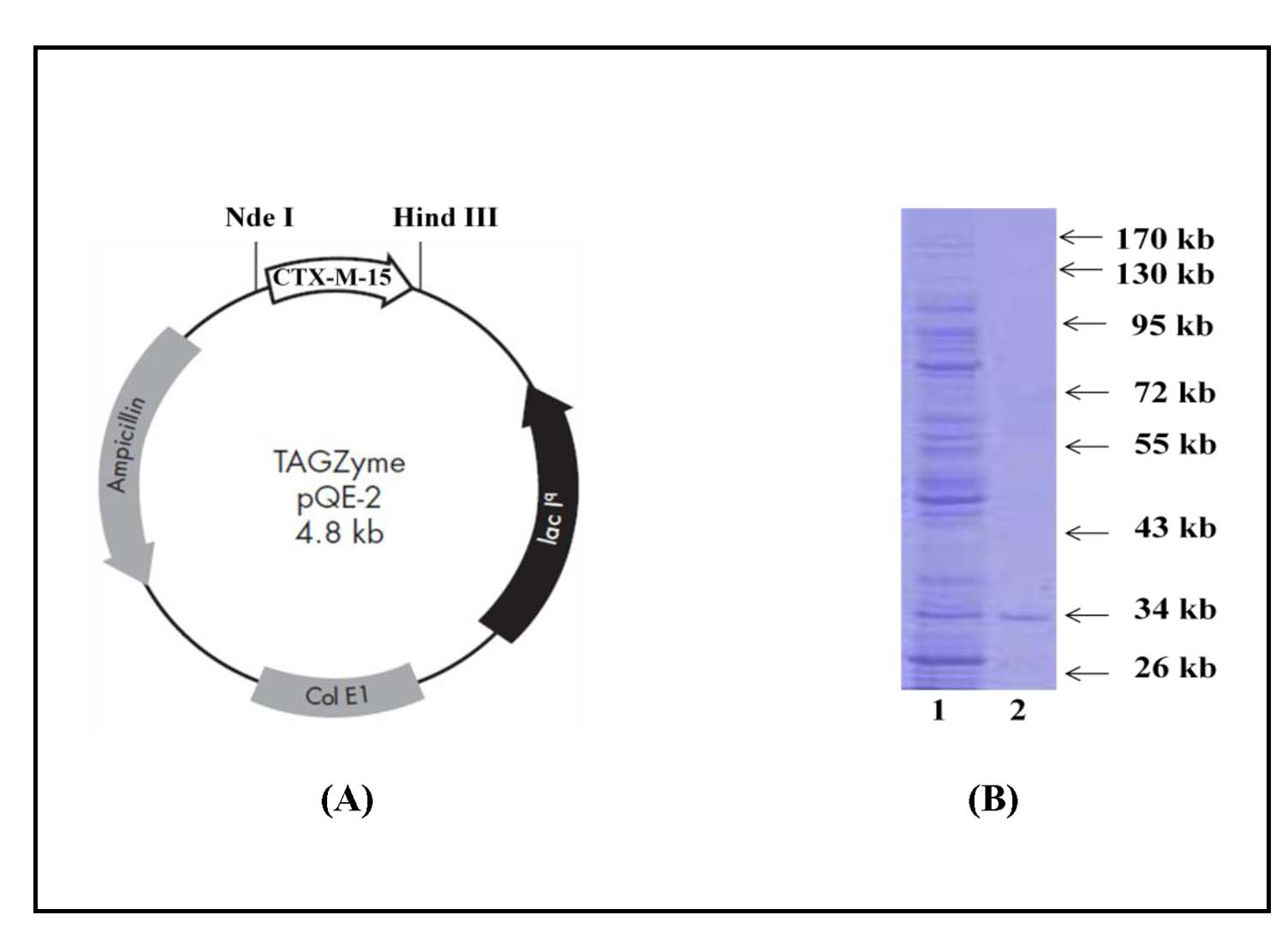

Supplement: Figure S1 — Cloning and purification of CTX-M-15. Panel (A) shows the vector used for cloning and expression of bla CTX-M-15 gene. Panel (B) is the SDS-PAGE of the purified CTX-M-15. Lane 1 and 2 are overexpressed total cell protein and purified protein, respectively. The single band represents molecular mass of 31 kDa. (TIF) [file pone.0056926.s001.tif]

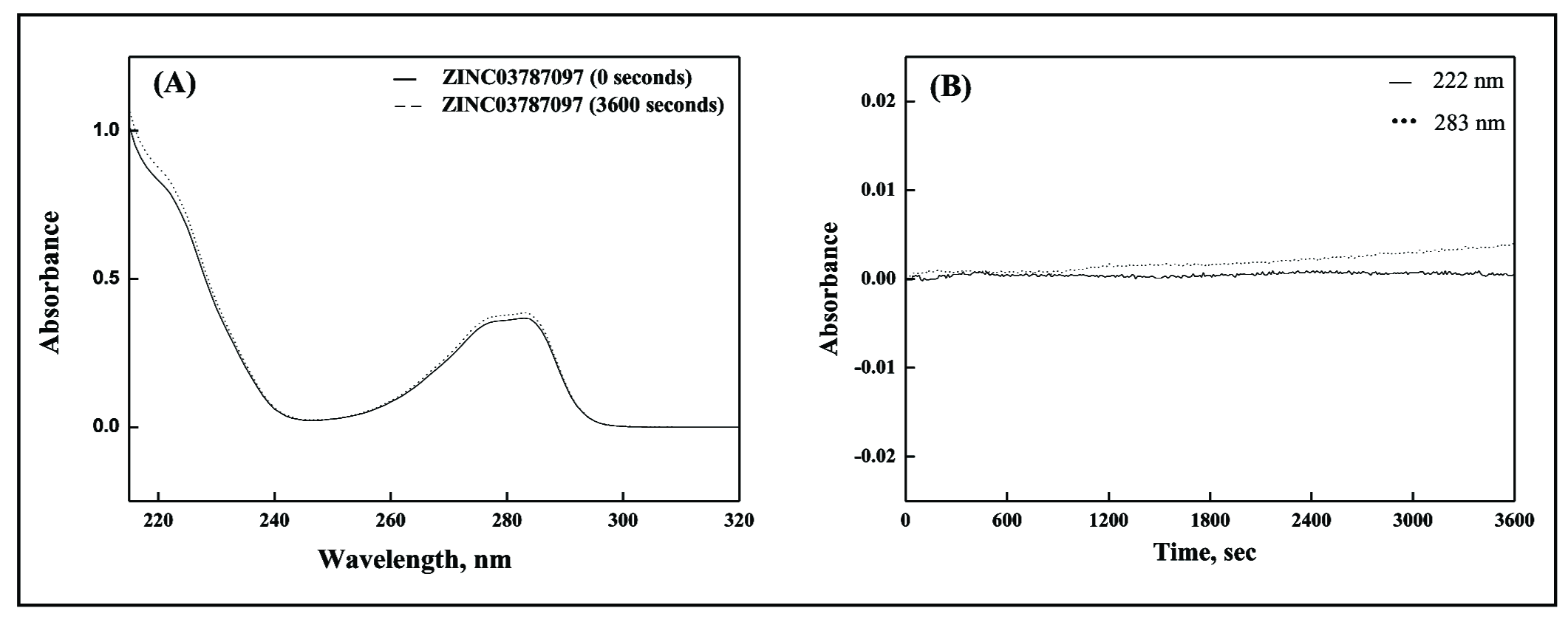

Supplement: Figure S4 — Stability of ZINC03787097 in the presence of CTX-M-15. Panel (A) shows the absorbance spectra of ZINC03787097 before and after 3600 seconds pre-incubation with CTX-M-15 at 30°C. Panel (B) shows the hydrolysis of ZINC03787097 by CTX-M-15 at 30°C monitored by measuring the change in absorbance at 283 nm and 222 nm. (TIF) [file pone.0056926.s004.tif]
